# Weak-coupling superconductivity in a strongly correlated iron pnictide

**DOI:** 10.1038/srep18620

**Published:** 2016-01-05

**Authors:** A. Charnukha, K. W. Post, S. Thirupathaiah, D. Pröpper, S. Wurmehl, M. Roslova, I. Morozov, B. Büchner, A. N. Yaresko, A. V. Boris, S. V. Borisenko, D. N. Basov

**Affiliations:** 1 Physics Department, University of California–San Diego, La Jolla, CA 92093, USA; 2 Leibniz Institute for Solid State and Materials Research, IFW, 01069 Dresden, Germany; 3 Max Planck Institute for Solid State Research, 70569 Stuttgart, Germany; 4 Solid State and Structural Chemistry Unit, Indian Institute of Science, Bangalore–560 012, India; 5 Department of Chemistry, Moscow State University, 119991 Moscow, Russia

## Abstract

Iron-based superconductors have been found to exhibit an intimate interplay of orbital, spin, and lattice degrees of freedom, dramatically affecting their low-energy electronic properties, including superconductivity. Albeit the precise pairing mechanism remains unidentified, several candidate interactions have been suggested to mediate the superconducting pairing, both in the orbital and in the spin channel. Here, we employ optical spectroscopy (OS), angle-resolved photoemission spectroscopy (ARPES), *ab initio* band-structure, and Eliashberg calculations to show that nearly optimally doped NaFe_0.978_Co_0.022_As exhibits some of the strongest orbitally selective electronic correlations in the family of iron pnictides. Unexpectedly, we find that the mass enhancement of itinerant charge carriers in the strongly correlated band is dramatically reduced near the Γ point and attribute this effect to orbital mixing induced by pronounced spin-orbit coupling. Embracing the true band structure allows us to describe all low-energy electronic properties obtained in our experiments with remarkable consistency and demonstrate that superconductivity in this material is rather weak and mediated by spin fluctuations.

Strong entanglement of various electronic and lattice degrees of freedom in the iron-based superconductors has become the *leitmotif* of recent condensed-matter research and has been identified with a vast variety of experimental probes^1^,^2^,^3^,^4^ and interpreted theoretically^5^,^6^. This inherent complexity arises from multiple partially filled Fe-3*d* orbitals simultaneously contributing to the low-energy quasiparticle dynamics and strongly affected by the Hund’s coupling correlations^7^. The orbitally selective nature of these interactions leads to a likewise orbitally selective bandwidth renormalization and relative energy shift of various bands with respect to each other and the Fermi level due to the pronounced particle-hole asymmetry of the electronic structure^8^. Such non-trivial interaction-induced modifications result in a singular Fermi-surface topology that is dramatically different from the predictions of theoretical calculations in both the number and the binding energy of the bands crossing the Fermi level^9^,^10^, strongly affecting low-energy electronic properties including superconductivity.

Here, we show that in the nearly optimally doped 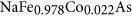 compound, low-energy orbitally selective renormalization of the band structure deviates markedly from the general experimental and theoretical renormalization trend in iron-based superconductors^11^. We find more than twice stronger than expected orbitally selective electronic correlations in the Fe-3*d*_xy_ band crossing the Fermi level near the Γ point of the Brillouin zone, similarly to FeTe_1−*x*_Se_*x*_^12^,^13^,^14^. However, in contrast to the observations in the latter compounds, the mass enhancement in the strongly correlated band of 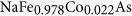 is drastically reduced near the Fermi level, where our relativistic *ab initio* calculations predict orbital mixing of the Fe-3*d*_*xy*_, 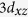, and 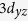 orbitals to occur due to spin-orbit coupling, leading to a pronounced modification of low-energy electronic properties. A simultaneous detailed analysis of the ARPES and OS data reveals remarkable consistency in the extracted itinerant properties of both the normal and the superconducting state. It allows us to conclude that the ubiquitous dichotomy between the coherent and incoherent far-infrared quasiparticle response observed in all iron-based superconductors^15^ is not dominated by the disparity in the mobilities of the hole and electron charge carriers but rather originates in the inelastic scattering from an intermediate bosonic excitation. Finally, all of the observed features in the far-infrared conductivity in the superconducting and normal state can be reproduced in an effective two-band Eliashberg model assuming relatively weak superconducting pairing with 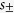 symmetry mediated by low-energy spin fluctuations with the frequency and temperature dependence observed in the same compound by inelastic neutron scattering^16^.

The effect of electronic correlations on the low-energy electronic structure of 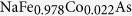 is illustrated in Fig. 1a–c. The general band structure of 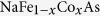 has been studied extensively and consists, similarly to most iron-based superconductors, of three hole bands at the Γ (Z) point of the Brillouin zone and two electron bands near the M point of the two-Fe Brillouin zone^17^,^18^ (the difference in the band structure at the Γ and Z points is minor, see Supplementary Information). Both of these sets of electronic dispersions are analyzed in Fig. 1a,b, respectively, and compared to the corresponding predictions of our band-structure calculations shown in Fig. 1c. In stark contrast to the prediction of the theory in Fig. 1c, only one hole band at the Γ point of 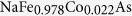 crosses the Fermi level, the remaining two are shifted to lower binding energies and terminate within about 20 meV below the Fermi level. These inner hole bands have a well-defined parabolic shape, as illustrated by the fits (lower white and red dashed lines) to the experimental band dispersions extracted from momentum-distribution curves at various binding energies (black solid lines). The comparison of the fitted effective masses to the theoretical band structure in Fig. 1c reveals a renormalization by a factor of 4.3 and 4.5 for the inner and middle hole bands of Fe-3*d*_*xz,yz*_ orbital character, respectively. The outer hole band of Fe-3*d*_*xy*_ orbital character in Fig. 1a shows a dramatic renormalization by a factor of more than 8 (blue dashed line), clearly demonstrating the existence of very strong and orbitally selective electronic correlations in 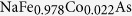, similar to 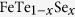
12,13,14 (the slight asymmetry of the photoemission intensity distribution in Fig. 1a does not affect our conclusions, see Supplementary Information). Such a strong enhancement of the effective quasiparticle mass is at odds with the predicted renormalization by a factor of only 3 to 4, identified in combined density-functional and dynamic mean-field theory calculations (DFT + DMFT) in ref. 11. Our analogous analysis of the experimental band structure of 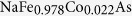 at the M point of the Brillouin zone (Fig. 1d) reveals two well-defined parabolic bands of electron character, consistent with the prediction of theory in Fig. 1c, with a very disparate effective-mass renormalization of about 2.2 and 5.7 for the inner and outer electron bands, respectively.

Quite surprisingly, the outer hole band’s dispersion reveals a departure from a parabolic shape (blue dashed line in Fig. 1a) upon approaching the Fermi level, clearly absent in the calculated band structure. To quantify this non-parabolicity, we extract the energy-dependent effective mass 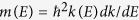, where 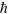 is the Planck constant, *k*—quasiparticle wave vector, and *E*—quasiparticle energy. For a parabolic dispersion 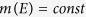. The resulting energy-dependent effective mass renormalization 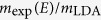 in all hole bands near the Γ point of the Brillouin zone 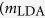 from Fig. 1c) is shown in Fig. 1e. It is clear that while the higher-energy (outside of the blue hatched area) effective mass of the outer hole band is simply renormalized from its theoretical counter part by a factor of 8, at low energies this is not the case.

This dramatic reduction of the effective mass at lower binding energies cannot be attributed solely to the effect of high-energy electronic correlations, which typically lead to an enhancement of the effective mass over the entire electronic band width. In the presence of orbitally selective renormalization the same is true for each band of a given orbital character. Our *ab initio* calculations (Fig. 1c) clearly show that the outer hole band has a well-defined 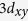 orbital character up to its termination. Recently, it has been demonstrated that spin-orbit coupling is not negligible in iron-based superconductors^19^. Provided that the hole bands of 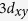 and 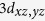 character are almost degenerate at the Γ point of the Brillouin zone, as can be seen in Fig. 1c, the existence of a sizable spin-orbit interaction would lead to the mixing of the orbital character in these three bands and potentially reduce the mass renormalization in the outer 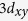 hole band due to a large admixture of more weakly renormalized 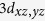 bands. The existence of such orbital mixing has been identified previously based on the polarization dependence of the photoemission intensity near the Γ (Z) point in ref. 17. Given the reduction of the effective mass in the outer hole band due to orbital mixing, one should expect a corresponding enhancement of the effective mass in the middle and/or inner hole bands due to the finite admixture of the heavier 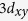 orbital. This effect, albeit rather subtle, can nevertheless be clearly identified in the second derivative of the experimental data in Fig. 1a upon close inspection (see Supplementary Information).

The aforementioned reduction of the effective mass due to orbital mixing does not rely on the existence of strong electronic correlations and should be observable already at the level of quasiparticle band masses. Indeed, Fig. 1d demonstrates that when spin-orbit coupling in this material is taken into account in a relativistic *ab initio* calculation (black solid lines), the dispersion of the hole bands at low energies shows a pronounced departure from that obtained in the non-relativistic calculation presented in Fig. 1c (grey solid lines). Both the reduction of the effective mass in the outer band and its enhancement in the middle and inner bands are apparent.

The complex modification of the low-energy electronic structure with respect to theory observed in our experiment has a profound effect on the itinerant properties of 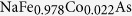. It strongly affects the analysis and interpretation of the OS data obtained on the same compound, as we will demonstrate below. The itinerant quasiparticle response can be extracted from the OS data by eliminating the contribution of all clearly identifiable interband transitions (green lines in Fig. 1f,g) from the optical conductivity 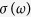 or, equivalently, dielectric function 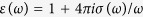 (see also Supplementary Information). The resulting itinerant optical response 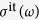, or 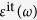, can be decomposed into a narrow (coherent) and a broad (incoherent) Drude-like contribution (blue and red lines in Fig. 1f,g). Such a decomposition is rather common in the iron-based superconductors and has been observed in essentially all known materials of this family^15^. At the same time, the electronic band structure of 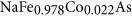 features three bands crossing the Fermi level, indicating the existence of a segregation of all free charge carriers into two *effective* electronic subsystems: one with a large and the other one with a small scattering rate (summarized in Fig. 1g).

It is tempting to assign the narrow Drude component to the combined response of charge carriers on the electron sheets of the Fermi surface (which in iron-based compounds have been found to have higher mobilities than hole carriers based on Hall and quantum-oscillation measurements^20^,^21^), and the broad one to the contribution of the carriers on the hole sheet of the Fermi surface. However, a direct comparison of the plasma frequencies of the two Drude terms extracted in our analysis of the optical conductivity (Fig. 1g) to those obtained from the ARPES data (as shown in Fig. 1a,b and detailed in the supplementary information) reveals that this assignment is incorrect: the plasma frequency of the hole sheet of the Fermi surface, 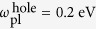, is substantially smaller than the total plasma frequency of the electron sheets, 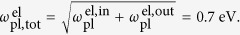 The reverse assignment (broad Drude component to electron charge carriers) cannot be reconciled with the negative Hall coefficient observed in this compound^22^. Additionally, a simple estimate of the electron mean free path due to elastic impurity scattering as 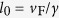, where *v*_F_ is the Fermi velocity and γ is the quasiparticle scattering rate obtained in our Drude-Lorentz analysis, leads to an unrealistic result. Taking 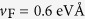 for the electron bands in Fig. 1b and 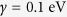 for the broad Drude component in Fig. 1f,g, one arrives at 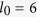 Å or less than two unit cells. Such a small mean free path is hard to justify in a nearly stoichiometric 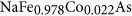. A similar estimate assuming 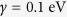 on the hole pocket 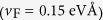 leads to 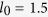 Å, or less than one unit cell. Analogous analysis of the broad Drude component in the optical conductivity of optimally doped 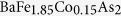 previously extracted a comparable mean free path assuming elastic scattering of holes^23^. These inconsistencies imply that the large scattering rate of the incoherent Drude component does not originate in the low mobility of one of the charge-carrier type but rather results from *inelastic* scattering of charge carriers (of either sign) from an intermediate boson. This conclusion is consistent with the association of the broad Drude component with electronic correlations based on the doping dependence of the optical conductivity in various iron-based compounds^24^,^25^.

The strength of electronic correlations is frequently estimated by comparing the total itinerant spectral weight (area under the itinerant optical conductivity curve) 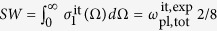, where 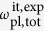 is the total itinerant plasma frequency, to the predictions of theoretical band-structure calculations^26^. While in particle-hole–symmetric single-band materials this approach provides a good estimate of effective-mass renormalization and thereby correlations, in iron-based superconductors the experimentally observed plasma frequency is modified from its theoretical prediction not only by orbitally selective band width renormalization but also intrinsic (see Supplementary Information) band shifts^9^, a process that in general conserves neither the Fermi wave vector nor the number of bands crossing the Fermi level, dramatically affecting the total plasma frequency. Therefore, the assessment of electronic correlations in such cases can only be reliably carried out based on the comparison of the complete low-energy experimentally observed *band structure* to its theoretically predicted counterpart.

Fortunately, even in such complicated cases the spectral-weight analysis of the itinerant optical response bears fruit. It is well-known that the total spectral weight of any system (including optical absorption in the ultraviolet and x-ray spectral range) is constant and does not depend on the details of the band structure: 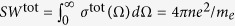 (in CGS units), where *n* is the electron density, *e* and *m*_*e*_ are the bare electron charge and mass, respectively. Quite similarly, the *itinerant* spectral weight (devoid of all interband transitions) is also constant, with *m*_*e*_ substituted by the quasiparticle band mass including renormalization at energies larger than the plasma frequency. Any low-energy mass renormalization due to, e.g., coupling to phonons or other bosonic excitations will not affect this spectral weight as long as its characteristic energy is smaller than the plasma frequency of free charge carriers. On the other hand, the low-energy band structure and the plasma frequency extracted from it will be affected by such renormalization. Therefore, the comparison of the total itinerant plasma frequency obtained from OS and ARPES data provides access to purely boson-exchange–induced effective mass renormalization even when it is not immediately apparent in the experimental band structure. In the present case of 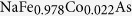 this renormalization amounts to 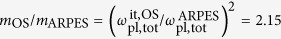 (see Fig. 1a,b,g). This is related to the strength of electron-boson couling *λ* via 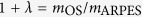, giving 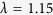.

Having established that only three out of five electronic bands predicted theoretically actually contribute to the itinerant carrier response of 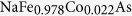 and that electrons are coupled to an intermediate boson with a coupling strength of 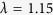, we now turn to the analysis of the superconducting state that develops in this electronic structure. Up to now, the character of superconductivity in optimally doped 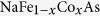  has remained unclear, with both weak and strong superconducting pairing suggested based on the ARPES measurements of the superconducting energy gap in refs. 17,18, respectively. This uncertainty can be eliminated based on the analysis of our bulk OS measurements in the far-infrared and THz spectral range, shown in Fig. 2. Panel 1a shows the temperature dependence of the relative change of the reflectance with respect to that at 30 K for several frequencies in the lowest THz spectral range. The normal-state trend for all frequencies is suddenly reversed near 18 K, which thus must be identified with the superconducting transition temperature, consistent with the previous ARPES measurements on the same samples^17^. The analysis of the real part of the optical conductivity in the far-infrared and THz spectral range reveals the quintessential feature of Cooper pairing—the dramatic suppression of the optical conductivity, with the missing area 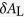 between the normal and the superconducting state at finite frequencies 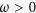 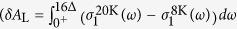, hatched area in Fig. 2b) transferred into the coherent response of the condensate at 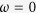 (ref. 27). Our analysis shows that this missing area corresponds to a London penetration depth 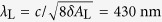, in a remarkable agreement with nuclear-magnetic–resonance and muon-spin–rotation measurements on this material^28^,^29^.

The vanishing of the optical conductivity in the superconducting state (blue solid line in Fig. 2b) further identifies the optical superconducting energy gap 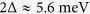 and the corresponding gap ratio 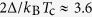, very close to the weak-coupling limit of the Bardeen-Cooper-Schrieffer theory of superconductivity^27^,^30^. These values are in a good agreement with those extracted from the ARPES data on the same single crystals^17^ and together provide compelling evidence for relatively weak-coupling superconductivity in 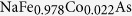.

The onset of absorption at 2Δ (blue solid line in Fig. 2b) is very sharp and has a Mattis-Bardeen shape, characteristic of a weakly coupled superconductor or that with large elastic impurity scattering^31^,^32^. Intriguingly, the frequency dependence of the optical conductivity beyond this initial increase is non-monotonic and reveals a quasilinear section, which deviates markedly from the Mattis-Bardeen shape and defines another energy scale, 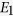. Such a quasilinear frequency behavior in the optical conductivity of iron-based superconductors is not unprecedented and has been previously observed in the response of optimally doped 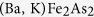
33,34 and attributed to the boson-mediated absorption in the clean limit (low impurity scattering) of a strongly coupled superconductor^35^.

It is quite clear that the combination of such very different features characteristic of a weakly and strongly coupled as well as clean- and dirty-limit superconductor in the optical response of the same material necessitates a comparison with a theoretical model that encompasses all of these limiting behaviors. The Eliashberg theory of superconductivity is perfectly suited to this task. The key input parameter of the theory is the spectrum of the bosonic excitation that mediates superconducting pairing and the overall electron-boson coupling strength. Spin dynamics in 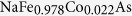 has been shown to be sensitive to superconductivity, as manifested in the formation of a resonance peak in the imaginary part of the spin susceptibility below *T*_c_ (refs. 16,36), while the electron-phonon interaction has been demonstrated to be insufficient to account for the elevated superconducting transition temperature of iron-based superconductors^37^. Therefore, we assume interband electron-electron interaction via the exchange of spin fluctuations with the experimentally obtained spectral shape in the normal and superconducting state (Fig. 2d) to mediate superconducting pairing in our model.

In the presence of the disparate features observed in the optical response of 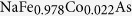 and an interband pairing interaction, the simplest, minimal, model would incorporate two effective electronic bands. The two-band Eliashberg formalism employed in our calculations has been detailed in ref. 38. We assume the symmetry of the superconducting order parameter to be of the *s*_±_-type. Given that the densities of states of all bands crossing the Fermi level are comparable and that all ARPES measurements find little variation in the size of the superconducting energy gap within and between the bands^17^,^18^, we assume two effective bands with isotropic superconducting energy gaps of the same magnitude and opposite sign, as well as equal densities of states. Elastic (impurity) *interband* scattering is neglected^35^. The plasma frequencies, *intraband* impurity scattering rates, and the coupling strength between the bands are the free parameters of the model tuned to fit the optical superconducting energy gap 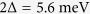, the superconducting transition temperature 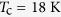, and the overall shape of the optical conductivity in the superconducting and normal state. Their optimal values obtained in the fit under the aforementioned constraints are indicated in Fig. 2c.

Figure 2c shows the result of our modeling (solid lines) overlaid on the experimental data in the superconducting (open circles) and normal (filled circles) state. It is immediately evident that *quantitative* agreement can be achieved for the parameters given in the panel. Quite remarkably, if not unprecedentedly, all of the transport parameters (the individual plasma frequencies of the bands and their *effective* impurity scattering rates) are in a very good agreement with the properties of the two effective itinerant electronic subsystems extracted in the Drude analysis of the conductivity data in Fig. 1g. Furthermore, the interband electron-boson coupling strength obtained from the fit 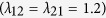 shows excellent agreement with the value obtained from the spectral-weight analysis above 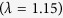.

Our theoretical analysis of the optical conductivity in the framework of an effective two-band Eliashberg model thus reconciles all of the characteristic energy scales and spectral features observed in 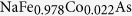. The sharp onset of absorption at 2Δ is dominated by the band with large impurity scattering. Energy scale 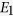 can be traced back to the onset of absorption in a clean superconductor via a Holstein-type process of breaking-up of a Cooper pair with a simultaneous creation of one spin-fluctuation quantum^39^,^40^. In our model the lowest energy cost of this process is 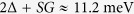, where 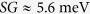 is the spin gap observed in the spin-fluctuation spectrum (see Fig. 2d), below which no spin fluctuations exist in the superconducting state. The quasilinear behavior above 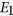 results from the quasilinear shape of the spin-fluctuation spectrum above the spin gap.

The remarkable agreement between different experimental probes and theoretical tools employed in this work demonstrates the importance of employing a realistic electronic band structure to interpret the low-energy electronic properties in such complex multiband systems as iron-based superconductors, if complete and consistent understanding of their properties is to be achieved. Our observation of a dramatic reduction of the effective mass in one of the electronic bands of 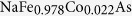, accompanied by relatively weak superconducting pairing, underlines the intimate interplay between different electronic degrees of freedom and the importance of spin-orbit coupling for the ground-state properties of the iron-based superconductors. It further shows that the modification of the orbital character of the bands crossing the Fermi level by means of intrinsic or extrinsic strain may allow for unprecedented control of low-energy electronic properties of these compounds, including superconductivity.

## Methods

Angle-resolved photoemission measurements were performed using synchrotron radiation (“1^3^–ARPES” end-station at BESSY) within the range of photon energies 20–90 eV and various polarizations on cleaved surfaces of high-quality single crystals of 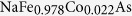. The overall energy and angular resolution were 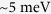 and 0.3°, respectively, for the low temperature measurements.

Optical-spectroscopy measurements were carried out in the THz and the far-infrared spectral range by means of the reflectance technique and from the far-inrared to the ultraviolet spectral range using spectroscopic ellipsometry. The air-sensitive samples of optimally doped 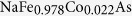 were cleaved before every measurement and loaded into the measurement chamber in the neutral atmosphere of argon gas, without exposing the sample to air at any time. High-accuracy absolute measurements of the sample’s reflectance were carried out using the gold-overfilling technique.

High-quality single crystals of superconducting 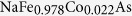 were synthesized by the self-flux technique and were characterized by x-ray diffraction, transport and magnetization measurements^41^. The latter revealed a superconducting transition temperature of about 18 K. The width of the superconducting transition was found to be less than 1 K, indicating very high homogeneity of the investigated samples.

Band structure calculations were performed for the experimental crystal structure of NaFeAs (ref. 42) using the PY LMTO computer code^43^.

## Additional Information

**How to cite this article**: Charnukha, A. *et al.* Weak-coupling superconductivity in a strongly correlated iron pnictide. *Sci. Rep.* **5**, 18620; doi: 10.1038/srep18620 (2015).

## Supplementary Material

Supplementary Information

## Figures and Tables

**Figure 1:**
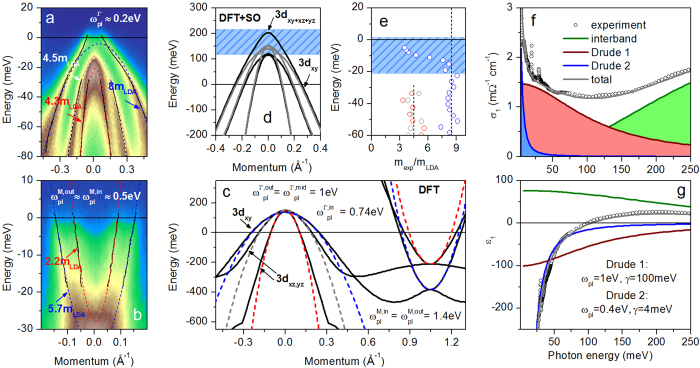
Low-energy electronic properties of NaFe_0.978_Co_0.022_As. (**a**,**b**) Low-energy electronic structure of 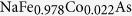 in the normal state near the Γ ((**a**) 30 K) and M ((**b**) 22 K) points of the Brillouin zone recorded using 20 eV and 25 eV photons linearly polarized perpendicular to and within the plane of incidence, respectively. The dispersion of all electronic bands observed in experiment (black solid lines) has been obtained using a multi-Lorentzian fit of the momentum-distribution curves. Effective-mass renormalization indicated in the panels has been calculated by comparing the low-energy parabolic fits to the electronic dispersions extracted from the experimental data (dashed lines in panels (**a**,**b**)) with those to the corresponding electronic bands predicted theoretically (dashed lines in panel (**c**)). (**c**) Theoretically predicted low-energy electronic band structure of NaFeAs in the 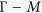 high-symmetry direction of the Brillouin zone (black solid lines) and low-energy parabolic fits to the dispersions near Γ and M (dashed lines). The colors of the dashed lines correspond to those in panels (**a,b**). Plasma frequencies of all bands crossing the Fermi level extracted from experimental data and predicted by theory are shown in panels (**a**–**c**). (**d**) Electronic band structure in the 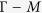 high-symmetry direction of the Brillouin zone near the Γ point obtained in a relativistic DFT calculation (black lines), which explicitly accounts for a finite spin-orbit coupling (its characteristic energy scale is shown as blue hatched area). The results of the corresponding non-relativistic calculation from panel **c** are shown for comparison (grey lines). (**e**) Energy dependence of the effective mass renormalization in the outer (blue open circles), middle (grey circles), and inner (red circles) hole band at the Γ point with respect to the corresponding low-energy quasiparticle masses extracted from the theoretically predicted band structure in panel (**c**) using parabolic fits. The energy scale affected by spin-orbit interaction is indicated as a blue hatched area. (**f**,**g**) Real part of the optical conductivity (**f**) and the dielectric function (**g**) of 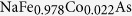 obtained in OS measurements (open circles) along with the results of a Drude-Lorentz dispersion analysis (blue and red lines/shaded areas and green line/shaded area for the two Drude terms and the total contribution of all interband transitions, respectively; gray solid lines indicate the sum of all terms). The parameters of the itinerant charge carrier response obtained by means of this analysis are summarized in panel (**g**).

**Figure 2:**
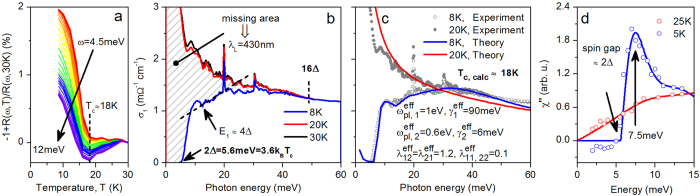
Superconductivity in nearly optimally doped NaFe_0.978_Co_0.022_As. (**a**) Relative change in the sample reflectance as a function of temperature for several frequencies in the THz spectral range. The transition into the superconducting state is evidenced by a marked increase in the sample reflectance below 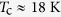 (vertical dashed line). (**b**) Real part of the itinerant THz and far-infrared conductivity of 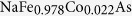 in the superconducting state at 8 K (blue solid line) and normal state at 20 and 30 K (red and black solid lines, respectively). The formation of Cooper pairs is manifested in the missing area under the conductivity curve in the superconducting vs normal state (hatched area), which amounts to a London penetration depth of 430 nm. Optical superconducting energy gap 2Δ is clearly visible as the energy at which the real part of the optical conductivity vanishes. The frequency-dependence of the optical conductivity above the initial onset of absorption in the superconducting state exhibits a quasilinear character, which defines an additional energy scale, 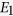 (for interpretation see text). Normal state optical conductivity is recovered at about 16Δ (vertical dashed line). (**c**) Comparison of the real part of the optical conductivity in the superconducting (8 K, gray open circles) and normal (20 K, gray filled circles) state obtained experimentally to the results of theoretical calculations in the framework of an effective two-band Eliashberg theory at 8 K (blue solid line) and 20 K (red solid line). Absorption below 2Δ in the superconducting state is due to thermally excited quasiparticles. All parameters of the theory are indicated in the panel. The initial sharp onset of absorption at 2Δ is assisted by elastic impurity scattering while the quasilinear frequency dependence of the optical conductivity at higher energies results from Holstein processes (breaking of a Cooper pair with a simultaneous creation of one or several quanta of the mediating boson). (**d**) The spectral function of the mediating boson in the normal (25 K) and superconducting (5 K) state used in the calculation (red and blue solid lines, respectively) and the imaginary part of the spin susceptibility obtained using inelastic neutron scattering on the same compound (open circles; data from ref. 16). Imaginary part of the spin susceptibility in the superconducting state clearly exhibits a resonance mode at 7.5 meV and a spin gap (SG) of 5.6 meV, approximately equal to the optical superconducting energy gap 2Δ in this material.
