# Supplementary material for: Weak-coupling superconductivity in a strongly correlated iron pnictide
Source: Sci Rep. 2016 Jan 5;6:18620. doi: 10.1038/srep18620 (PMC4700462; doi:10.1038/srep18620)
Supplement: Supplementary Information [file srep18620-s1.pdf]

# Weak-coupling superconductivity in a strongly correlated iron pnictide

A. Charnukha[1,2,3], K. W. Post[1], S. Thirupathaiah[2,4], D. Pröpper[3], S. Wurmehl[2], M. Roslova[2,5], I. Morozov[2,5], B. Büchner[2], A. N. Yaresko[3], A. V. Boris[3], S. V. Borisenko[2] & D. N. Basov[1]

[1]Physics Department, University of California–San Diego, La Jolla, CA 92093, USA, [2]Leibniz Institute for Solid State and Materials Research, IFW, 01069 Dresden, Germany, [3]Max Planck Institute for Solid State Research, 70569 Stuttgart, Germany, [4]Solid State and Structural Chemistry Unit, Indian Institute of Science, Bangalore–560 012, India, [5]Department of Chemistry, Moscow State University, 119991 Moscow, Russia. [†] E-mail: acharnukha@ucsd.edu.

## I. SPIN-ORBIT COUPLING IN THE LOW-ENERGY ELECTRONIC STRUCTURE OF $NaFe_{0.978}Co_{0.022}As$ NEAR THE Γ POINT

In order to confirm that the slight anisotropy of the photoemission intensity in the energy-momentum cut shown in Fig.1a of the main text does not affect the conclusions of our study we have fitted the left and right branches of the outer band's dispersion independently and compared these fits to the results of the simultaneous fit of both branches presented in the text. Only the data points at high enough binding energies (unaffected by the reduction in the effective mass due to the spin-orbit coupling) were used for all fits (red and green filled circles); the entire dispersion of the band is shown for completeness (grey empty circles).

The results of this comparison are shown in Fig. S1a. The red and green solid lines are the parabolic dispersions obtained from the fit of the data points of the corresponding color (only the left and right branches of the parabola were used in the fit of the red and green data points, respectively). It is clear that the independent fit of the right (green solid line) branch gives results very similar to the simultaneous fit of both branches (blue solid line). The independent fit of the left branch deviates somewhat from the simultaneous fit, albeit it provides only a marginal improvement in the fit's figure of merit. To quantify the effect of this uncertainty on the extracted renormalization of the effective mass we have calculated the latter using the formula given in the main text, $m(E) = \hbar^2 k(E) dk/dE$, independently for the left and right branches. The resulting energy-dependent effective mass is shown in Fig. S1b for the left (red open circles) and right (green open circles) branches of the dispersion along with their average (blue open circles) plotted in Fig.1e of the main text. It can be seen that even the lowest value of the renormalization corresponding to

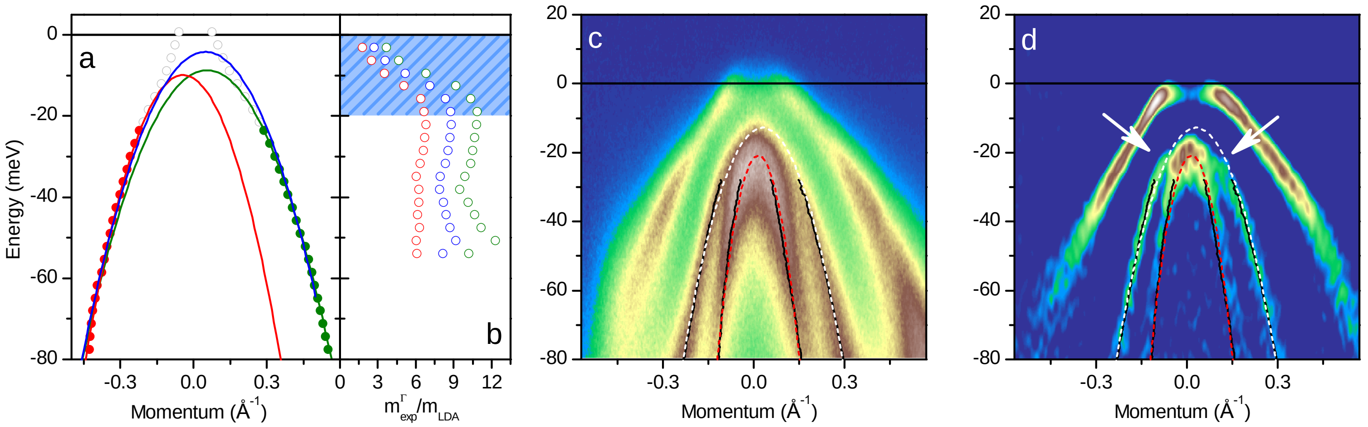


**Figure S1 | Details of the low-energy electronic band structure of $NaFe_{0.978}Co_{0.022}As$ near the Γ point. a,** Energy dispersion of the outer hole band near the Γ point of the Brillouin zone (circles) extracted from the maximum of the quasiparticle peak in the momentum-distribution curves overlaid with the simultaneous parabolic fit of both the left and right branches (blue solid line) as well as independent parabolic fits of the left (red solid line) and right (green solid line) branches. The data used for the fitting are shown as filled circles of the corresponding color. Grey open circles show the energy dispersion up to the Fermi level. **b,** Renormalization of the effective mass extracted independently from the left (red open circles) and right (green open circles) branches of the dispersion of the outer hole band, as well as their average, as shown in Fig.1e of the main text. **c,d,** Energy-momentum cut along the Γ-M direction of the Brillouin zone plotted in Fig.1a of the main text (panel **c**) and the second derivative of this photoemission intensity distribution taken in the direction of energy (panel **d**). Solid and dashed lines are as in Fig.1a of the main text. White arrows indicate the flattening of the energy dispersion of the middle hole band due to orbital mixing induced by spin-orbit coupling.

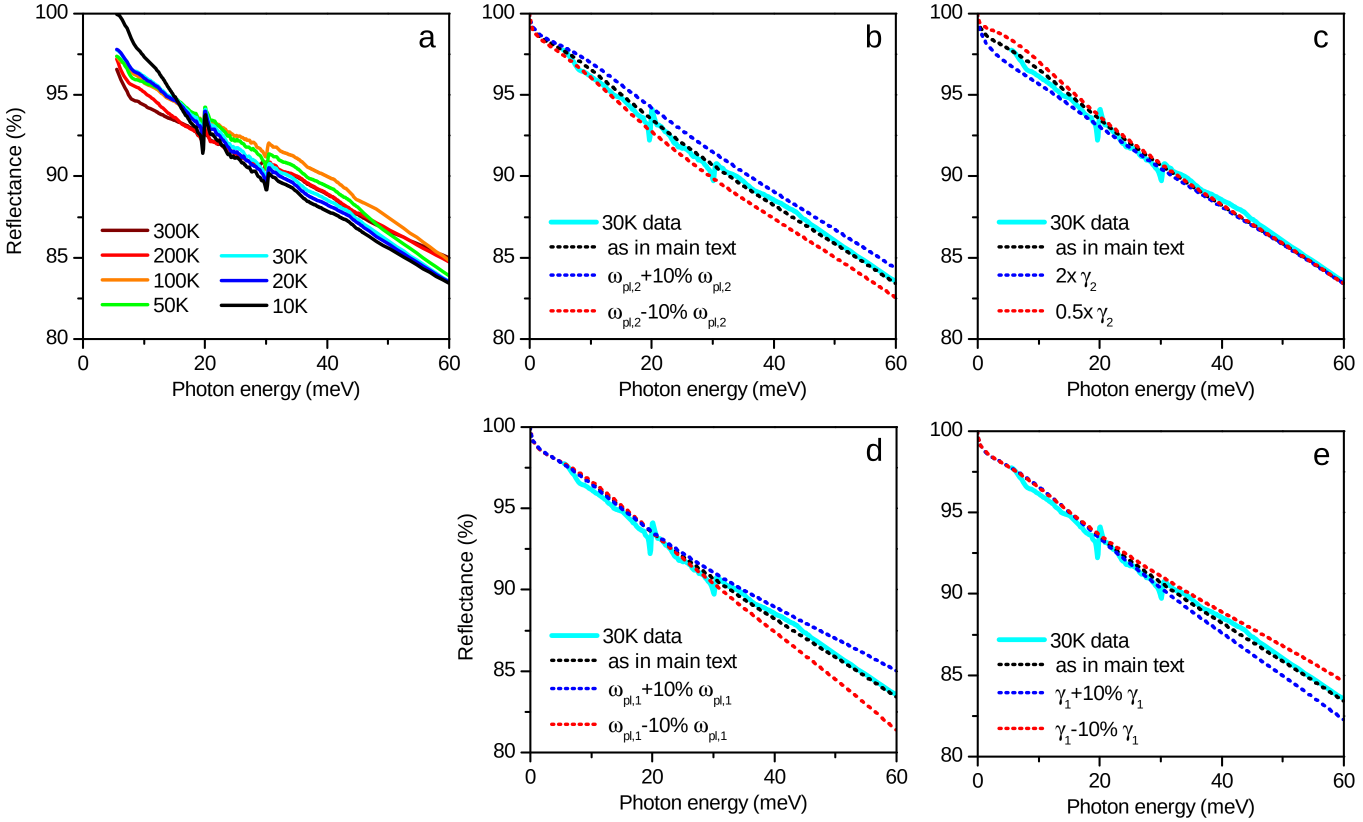


**Figure S2 | Details of the far-infrared data and analysis. a,** Photon-energy dependence of the far-infrared reflectance at various temperatures. **b–e,** Analysis of the reflectance at 30 K (cyan solid line) in the framework of the two-Drude model (dotted lines). Black dotted line shows the reflectance corresponding to the best-fit parameters of the two Drude terms, as specified in Fig.1g of the main text. Red and blue dotted lines demonstrate the effect of detuning these parameters up (blue lines) or down (red lines) from the best-fit values, as detailed in the figure legends. The high-frequency dielectric function used in this model was taken to be $\varepsilon_\infty = 60$, determined by the contribution of the interband transitions at higher energies.

the independent fit of the left branch is still significantly larger than the renormalization of all the other bands and the largest in the family of the high-temperature iron-pnictide superconductors. In view of the independent fits shown in Fig. S1 it is more likely that the true value of the renormalization is closer to the average than to the lowest value. Similar results can be obtained through the analysis of quasiparticle dispersion in the symmetrized (with respect to zero wave vector) data.

Figures S1c,d demonstrate the flattening of the middle hole band's dispersion (white arrows) near its top due to the orbital-mixing effect induced by the spin-orbit coupling.

## II. INHERENT CHARACTER OF THE ORBITALLY SELECTIVE BAND SHIFTS IN THE ELECTRONIC STRUCTURE OF $NaFe_{0.978}Co_{0.022}As$

In this section we would like to briefly substantiate the bulk-related character of the orbitally selective band shifts or, equivalently, disprove their surface-related origin. Orbitally selective band width renormalization and band shifts are generic to iron-based superconductors, have been observed in essentially all materials of this family [S 1,1–9], and suggested to result from orbital blocking in the presence of strong Hund's coupling [S 10] and a pronounced particle-hole asymmetry [S 11], respectively. This renormalized and band-shifted electronic structure, as well as the superconducting energy gap that develops on it, observed via ARPES has been found to agree remarkably well with many bulk-sensitive techniques, strongly suggesting that the electronic band structure of the surface layer in these compounds is reflective of that in the bulk. The only exception to this rule is the 1111 type of compounds, in which the presence of both bulk- and surface-related states in the photoemission signal has been found [S 12–21], making the extraction of the inherently bulk electronic structure challenging. Recently, a detailed ARPES work succeeded in disentangling the bulk and surface contributions to the photoemission signal and demonstrated that the bulk electronic structure features the same orbitally selective band width renormalization and band shifts as observed in other classes of iron-based superconductors [S 11], proving its generic and inherent character. Finally, it is well-known that 111-type materials cleave between the two layers of the intercalant, which results in a non-polar surface. The equivalence of the bulk and surface electronic states has been further

demonstrated in an explicit density-functional calculation [S 22].

## III. DETAILS OF THE FAR-INFRARED DATA AND ANALYSIS

Here we would like to demonstrate the degree of uncertainty in the extracted parameters of the free-charge-carrier response shown in Fig.1g that the finite signal-to-noise ratio of and the uncovered lowest-frequency range in the experimental data allow. We do so by comparing the optical response corresponding to the best fit shown in Figs.1f,g with that derived from the Drude-term parameters slightly detuned from those optimal values. In order to eliminate the integral transformation via Kramers-Kronig relations and to emphasize the functional shape of the reflectance assumed in the extrapolation region at lowest frequencies for Kramers-Kronig analysis, we demonstrate these trends in the raw far-infrared reflectance data. The latter are shown for different temperatures between 10 K and 300 K in Fig. S2a. Figures S2b–e show the reflectance obtained in our experiment as a function of photon energy at 30 K overlaid with that produced by assuming two Drude terms ($\omega_{\text{pl},1}, \gamma_1$; $\omega_{\text{pl},2}, \gamma_2$) with the parameters specified in the figure legends. The high-frequency dielectric function is taken to be $\varepsilon_\infty = 60$, determined by the contribution of the interband transitions at higher energies. The default (best-fit) parameters of the free-charge-carrier response referred to in these panels are those specified in Fig.1g of the main text.

## IV. DETERMINATION OF BAND-SPECIFIC PLASMA FREQUENCIES BASED ON ARPES DATA

In optimally doped $NaFe_{0.978}Co_{0.022}As$ the Fermi surface consists of three sheets: two electonlike in the corners of the Brillouin zone and one holelike at the center [S 23]. It is well-known and a generic feature of iron-based superconductors that the bands generating the electron sheets are only weakly dispersive in the out-of-plane direction, giving rise to two quasi-cylindrical sheets of the Fermi surface with relatively small corrugations [S 24]. Therefore, we can approximate these two sheets by two identical ideal cylinders with their radii equal to the average of the long and the short half-axis of the ellipsoids giving rise to the corrugation. The electronic band generating the hole sheet of the Fermi surface also disperses rather weakly along the Γ-Z direction of the Brillouin zone, as shown in Fig. S3: the Fermi wave vector of this band remains within 0.07–0.09 $\text{Å}^{-1}$ between Γ and Z. Therefore, the Fermi-surface sheet generated by this band can likewise be approximated by a cylinder with a radius of about 0.08 $\text{Å}^{-1}$. Thus the task of calculating the plasma frequency has been reduced to a two-dimensional problem. In general, the plasma frequency of free charge carriers can be obtained from the semi-classical expression for the dielectric function [S 25] assuming the intraband case and low temperatures and is given by the following expression (in SI units):

$$\omega_{\text{pl},\sigma}^2 = \frac{e^2}{8\pi^3\hbar^2\varepsilon_0}\sum_n \int \left[\frac{dE_{n\mathbf{k}}}{dk_\sigma}\right]^2 \delta(E_{n\mathbf{k}} - E_\text{F})d^3\mathbf{k}, \tag{1}$$

where $\sigma = x, y, z$ enumerates the coordinate axes, $n$ is the band index, $\delta(x)$ is Dirac's delta function, $E_{n\mathbf{k}}$ is the energy dispersion of the $n$th band, $E_\text{F}$ is the Fermi energy, $e$ is the electron charge, $\hbar$ is the reduced Planck constant, and $\varepsilon_0$ is the vacuum permittivity. Assuming our two-dimensional approach and the tetragonal symmetry of $NaFe_{0.978}Co_{0.022}As$, $\omega_{\text{pl},z} = 0$ and $\omega_{\text{pl},x} = \omega_{\text{pl},y} = \omega_\text{pl}$.

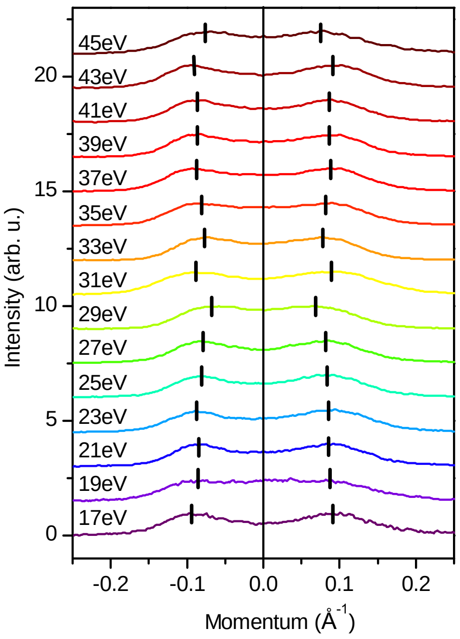


**Figure S3 | Out-of-plane dispersion of the hole band along the Γ-Z direction in the Brillouin zone.** Momentum-distribution curves (MDCs) at near-normal photoemission (0 momentum corresponds to normal emission) averaged in a 5 meV energy window around the Fermi level for various energies of incident photons. The data were recorded in the normal state of $NaFe_{0.95}Co_{0.05}As$ at 27 K. The MDCs are displaced vertically by a constant of 1.5. Black vertical lines indicate the position of the Fermi wave vector extracted as the maximum of the quasiparticle peak for every MDC.

Thus only one quantity $\omega_{\rm pl}$ needs to be calculated for each band. In the two-dimensional case and for an isotropic parabolic band with an effective band mass $m_{\rm eff}$ $[E_{\mathbf{k}} = \hbar^2\mathbf{k}^2/(2m_{\rm eff})]$ Eq. 1 can be rewritten as a simple and familiar expression:

$$\omega_{\rm pl}^2 = \frac{N_{\rm 2D}(E_{\rm F})e^2}{m_{\rm eff}\varepsilon_0}, \tag{2}$$

where $N_{\rm 2D}(E_{\rm F}) = \frac{k_{\rm F}^2}{(2\pi)^2}\frac{2\pi}{c}$ is the density of states at the Fermi level for an isotropic parabolic band in two dimensions, $k_{\rm F}$ is the Fermi wave vector, and $c$ is the out-of-plane lattice constant. Thus the calculation of the band-specific plasma frequencies is reduced to the determination of the Fermi wave vector and the quasiparticle effective mass for each band, which can easily be done based on the photoemission intensity distributions near the Fermi level presented in Figs.1a,b. As already mentioned above and shown in Fig. S3, the Fermi wave vector of the hole band is about 0.08 Å$^{-1}$, and its effective mass is found to be 3.83 $m_{\rm e}$ (where $m_{\rm e}$ is the bare electron mass), which results in a plasma frequency of $\omega_{\rm pl}^{\rm hole,1} = 0.23$ eV. The Fermi wave vectors and effective masses of the electron bands at the M-point are 0.086 Å$^{-1}$, 0.92 $m_{\rm e}$ and 0.153 Å$^{-1}$, 2.34 $m_{\rm e}$ for the inner and the outer band, respectively. These values result in almost identical plasma frequencies of $\omega_{\rm pl}^{\rm el,in} = 0.5$ eV and $\omega_{\rm pl}^{\rm el,out} = 0.56$ eV, respectively.

## References


S1. Borisenko, S. V. *et al.* Superconductivity without Nesting in LiFeAs. *Phys. Rev. Lett.* **105**, 067002 (2010).

S2. Sudayama, T. *et al.* Doping-Dependent and Orbital-Dependent Band Renormalization in $Ba(Fe_{1-x}Co_x)_2As_2$ Superconductors. *J. Phys. Soc. Jpn.* **80**, 113707 (2011).

S3. Ding, H. *et al.* Electronic structure of optimally doped pnictide $Ba_{0.6}K_{0.4}Fe_2As_2$ : a comprehensive angle-resolved photoemission spectroscopy investigation. *J. Phys.: Condens. Matter* **23**, 135701 (2011).

S4. Cui, S. T. *et al.* Evolution of the band structure of superconducting NaFeAs from optimally doped to heavily overdoped Co substitution using angle-resolved photoemission spectroscopy. *Phys. Rev. B* **86**, 155143 (2012).

S5. Yi, M. *et al.* Observation of Temperature-Induced Crossover to an Orbital-Selective Mott Phase in $A_xFe_{2-y}Se_2$ ($A$ =K,Rb) Superconductors. *Phys. Rev. Lett.* **110**, 067003 (2013).

S6. Maletz, J. *et al.* Unusual band renormalization in the simplest iron-based superconductor $FeSe_{1-x}$. *Phys. Rev. B* **89**, 220506 (2014).

S7. Lee, G. *et al.* Orbital Selective Fermi Surface Shifts and Mechanism of High $T_c$ Superconductivity in Correlated $A$FeAs ($A$ =Li, Na). *Phys. Rev. Lett.* **109**, 177001 (2012).

S8. Evtushinsky, D. V. *et al.* Strong electron pairing at the iron $3d_{\rm xz,yz}$ orbitals in hole-doped $BaFe_2As_2$ superconductors revealed by angle-resolved photoemission spectroscopy. *Phys. Rev. B* **89**, 064514 (2014).

S9. Brouet, V. *et al.* Large Temperature Dependence of the Number of Carriers in Co-Doped $BaFe_2As_2$. *Phys. Rev. Lett.* **110**, 167002 (2013).

S10. Yin, Z. P. , Haule, K. & Kotliar, G. Kinetic frustration and the nature of the magnetic and paramagnetic states in iron pnictides and iron chalcogenides. *Nature Mater.* **10**, 932–935 (2011).

S11. Charnukha, A. *et al.* Interaction-induced singular Fermi surface in a high-temperature oxypnictide superconductor. *Sci. Rep.* **5**, 10392 (2015).

S12. Lu, D. H. *et al.* Electronic structure of the iron-based superconductor LaOFeP. *Nature* **455**, 81–84 (2008).

S13. Liu, H.-Y. *et al.* Pseudogap and Superconducting Gap in $SmFeAs(O_{1-x}F_x)$ Superconductor from Photoemission Spectroscopy. *Chin. Phys. Lett.* **25**, 3761 (2008).

S14. Kondo, T. *et al.* Momentum Dependence of the Superconducting Gap in $NdFeAsO_{0.9}F_{0.1}$ Single Crystals Measured by Angle Resolved Photoemission Spectroscopy. *Phys. Rev. Lett.* **101**, 147003 (2008).

S15. Liu, C. *et al.* Electronic properties of iron arsenic high temperature superconductors revealed by angle resolved photoemission spectroscopy (ARPES). *Phys. C: Supercond.* **469**, 491–497 (2009).

S16. Lu, D. *et al.* ARPES studies of the electronic structure of LaOFe(P, As). *Phys. C: Supercond.* **469**, 452–458 (2009).

S17. Liu, H. *et al.* Unusual Electronic Structure and Observation of Dispersion Kink in CeFeAsO Parent Compound of FeAs-based Superconductors. *Phys. Rev. Lett.* **105**, 027001 (2010).

S18. Liu, C. *et al.* Surface-driven electronic structure in LaFeAsO studied by angle-resolved photoemission spectroscopy. *Phys. Rev. B* **82**, 075135 (2010).

S19. Yang, L. X. *et al.* Surface and bulk electronic structures of LaFeAsO studied by angle-resolved photoemission spectroscopy. *Phys. Rev. B* **82**, 104519 (2010).

S20. Yang, L. *et al.* Electronic structure of SmOFeAs. *J. Phys. Chem. Solids* **72**, 460–464 (2011).

S21. Nishi, I. *et al.* Angle-resolved photoemission spectroscopy study of $PrFeAsO_{0.7}$: Comparison with LaFePO. *Phys. Rev. B* **84**, 014504 (2011).

S22. Lankau, A. *et al.* Absence of surface states for LiFeAs investigated using density functional calculations. *Phys. Rev. B* **82**, 184518 (2010).

S23. Thirupathaiah, S. *et al.* Weak-coupling superconductivity in electron-doped $NaFe_{0.95}Co_{0.05}As$ revealed by ARPES. *Phys. Rev. B* **86**, 214508 (2012).

S24. Mazin, I. I. & Schmalian, J. Pairing symmetry and pairing state in ferropnictides: Theoretical overview. *Phys. C* **469**, 614–627 (2009).

S25. Yu, P. Y. & Cardona, M. *Fundamentals of Semiconductors* (Berlin: Springer, 2005).
